# Genomic trajectories of colorectal cancer with choroidal metastasis: longitudinal insights from tissue and liquid biopsy via next-generation sequencing

**DOI:** 10.3389/fgene.2025.1632580

**Published:** 2025-08-06

**Authors:** Alessandro Ottaiano, Luisa Circelli, Carmine Picone, Monica Ianniello, Raffaella Ruggiero, Roberto Sirica, Mariachiara Santorsola, Anna Chiara Carratù, Nadia Petrillo, Gaetana Cerbone, Francesco Sabbatino, Massimiliano Berretta, Guglielmo Nasti, Giovanni Savarese

**Affiliations:** ^1^ Istituto Nazionale Tumori di Napoli, IRCCS “G. Pascale”, Napoli, Italy; ^2^ AMES, Centro Polidiagnostico Strumentale srl, Casalnuovo diNapoli, Italy; ^3^ Division of Medical Genetics, “S.G. Moscati” Hospital, Avellino, Italy; ^4^ Medical Oncology, Department of Medicine, Surgery and Dentistry, University of Salerno, Baronissi, Italy; ^5^ Department of Clinical and Experimental Medicine, University of Messina, Messina, Italy

**Keywords:** choroidal metastasis, next-generation sequencing, circulating tumour DNA, tumour heterogeneity, colorecal cancer

## Abstract

Colorectal cancer (CRC) is a leading cause of cancer-related death, with metastases typically involving the liver, lungs, and peritoneum. Choroidal metastases are extremely rare. We report a case of metastatic CRC with choroidal involvement, characterized by longitudinal genomic profiling using the TruSight Oncology 500^®^ assay. A 66-year-old man with rectosigmoid junction CRC initially showed *RAS*/*BRAF* wild-type status, microsatellite stability (MSS), and a moderate tumor mutational burden (TMB: 7.1 mutations/Mb) on the primary tumor. First-line chemotherapy combined with anti-EGFR therapy was initiated for synchronous liver metastases. Upon the development of visual symptoms, imaging confirmed choroidal metastasis. Circulating tumor DNA (ctDNA) analysis revealed persistence of the *TP53* p.E286K mutation and marked clonal evolution. Newly emerged Tier IA alterations included *EGFR* amplification and *JAK2* p.V617F mutation, alongside multiple Tier IIC and IID variants absent in the primary tumor. The ctDNA also revealed a hypermutated phenotype (TMB: 44.9 mutations/Mb). To our knowledge, this is the first report integrating both tissue- and liquid-based NGS in a CRC case with ocular metastasis. These findings highlight the value of comprehensive genomic monitoring in metastatic CRC and may offer insights into the molecular landscape of rare metastatic sites such as the choroid.

## Introduction

Colorectal cancer (CRC) ranks as the third most commonly diagnosed malignancy in both men and women and it remains the second leading cause of cancer-related mortality overall (Pinheiro et al., 2024; Alzahrani et al., 2021). Approximately 20% of patients with CRC present with distant metastases at the time of diagnosis, and an additional 30% develop metastatic disease as their illness progresses. The liver, lungs, peritoneum, and lymph nodes represent the most common sites of metastasis in CRC (Marcellinaro et al., 2023). In contrast, ocular dissemination, particularly to the choroid, is exceptionally rare and remains poorly characterized from both clinical and molecular perspectives (Demir et al., 2024). The choroid is a highly vascularized layer of the eye, located between the retina and the sclera, which makes it particularly susceptible to hematogenous dissemination of cancer cells. Nevertheless, metastatic involvement of the choroid is uncommon, most frequently arising from primary tumors of the breast and lung (Shields et al., 2023). Gastrointestinal malignancies account for only a small fraction, approximately 4%, of all choroidal metastases, with colorectal primaries being particularly rare (Khawaja et al., 2015). Due to its rarity, choroidal metastasis from CRC has been documented in only a few isolated cases. A recent literature review reported just 25 such cases (Demir et al., 2024), often in the context of widespread metastatic disease, suggesting ocular involvement as a late event in tumor progression.

Advancements in next-generation sequencing (NGS) technologies and liquid biopsy platforms have enabled real-time tracking of tumor evolution and dissemination. The analysis of circulating tumor DNA (ctDNA) provides a minimally invasive window into the dynamic clonal architecture of cancer, including metastatic lesions that are otherwise inaccessible to tissue biopsy (Zalis et al., 2024). The TruSight Oncology 500 (TSO500) assay is a comprehensive genomic profiling tool capable of detecting somatic mutations, copy number alterations, microsatellite instability (MSI), and tumor mutational burden (TMB) across a wide panel of cancer-related genes, using both tissue and plasma-derived DNA (Esposito Abate et al., 2024; Al-Kateb et al., 2025).

In this report, we present a case of metastatic CRC with choroidal involvement, for which genomic profiling was performed at two critical timepoints: first, on a tissue biopsy of the primary tumor; and later, on a liquid biopsy (ctDNA) obtained during systemic dissemination involving the liver and the choroid.

To our knowledge, this is the first reported case to describe the genomic evolution of a CRC with ocular dissemination using an integrated tissue- and liquid-based NGS approach, providing insights into the molecular trajectory of a tumor progressing toward choroidal metastatization.

## Case presentation

**FIGURE 1:**
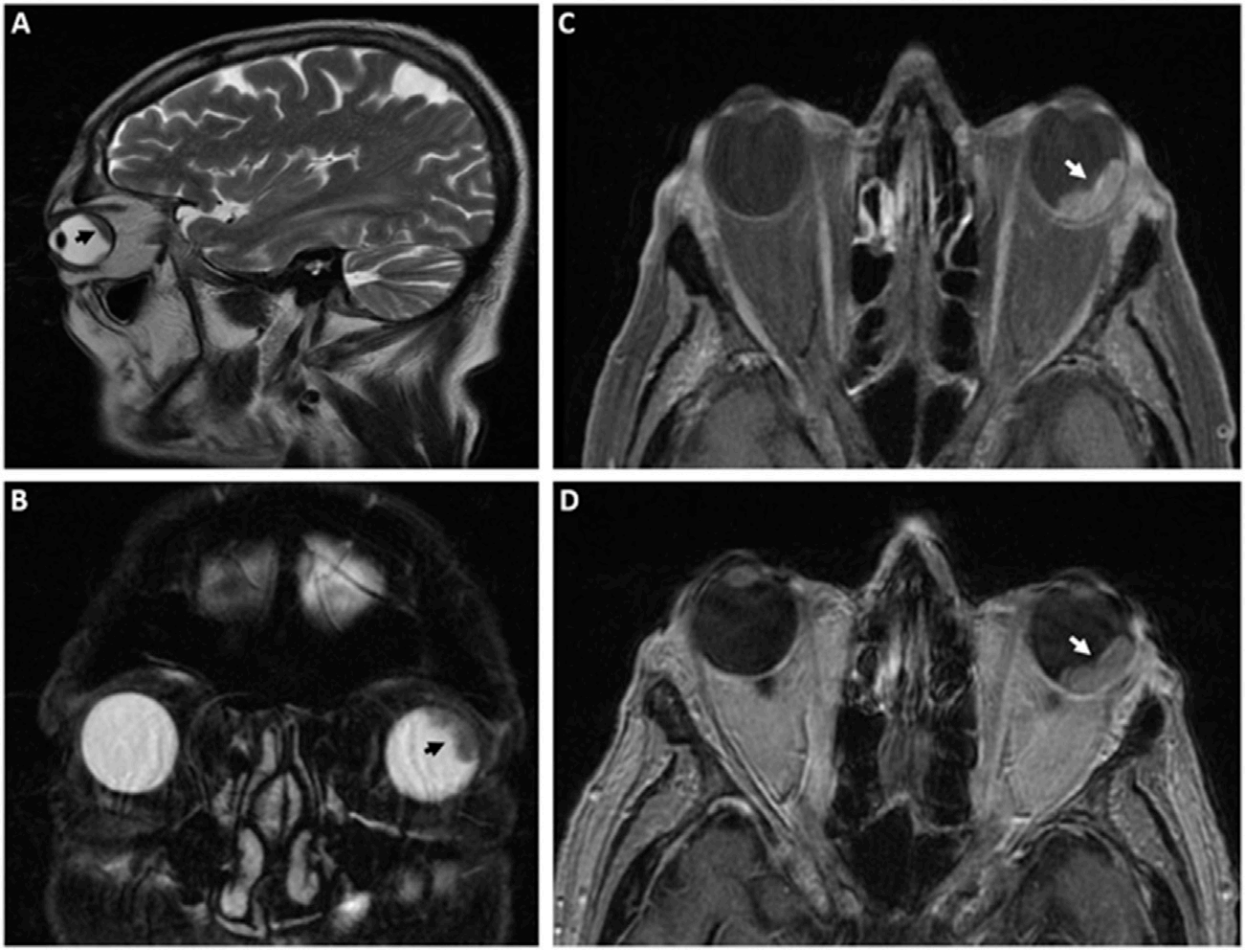
Magnetic resonance imaging (MRI) of the choroidal lesion. A lobulated area of altered signal intensity is observed along the supero-external aspect of the left ocular globe, measuring approximately 19 mm at its largest diameter, with pathological contrast enhancement. MRI sequences include sagittal **(A)** and coronal **(B)** T2-weighted images, axial T1-weighted fat-saturated **(C)**, and axial T1-weighted **(D)** images. The choroidal lesion is indicated by white or black arrows.

**FIGURE 2:**
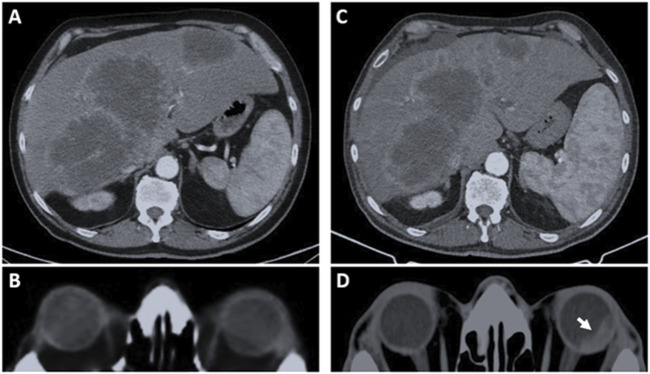
Selected computed tomography (CT) images before and after first-line therapy. Panels A and B refer to the baseline CT assessment (October 2024). **(A)** Multiple hypodense hepatic lesions with peripheral contrast enhancement are evident in both lobes. The largest lesion, measuring approximately 13 cm, is located in segments VII–VIIs. **(B)** The cranial-encephalic evaluation shows no evidence of choroidal involvement, and the patient reported no ocular symptoms at that time. **(C)** Post-treatment reassessment (March 2025) shows substantial stability of hepatic disease, but reveals a newly detected choroidal lesion in the left eye as indicated by the white arrow **(D)**, with an estimated extent of approximately 18 mm.

MS, a 66-year-old man, presented in October 2024 with abdominal discomfort, progressive dyspepsia, and a general decline in clinical condition. A contrast-enhanced total-body computed tomography (CT) scan performed on October 28 revealed multiple hepatic lesions, raising suspicion of metastatic disease. Serum levels of CEA and CA19.9 tumor markers were elevated. Subsequent colonoscopy identified an ulcerated mass at the rectosigmoid junction, and histopathological analysis of biopsy specimens confirmed a moderately differentiated adenocarcinoma. Initial molecular characterization of the primary tumor demonstrated wild-type status for both *RAS* and *BRAF*, preserved dihydropyrimidine dehydrogenase (DPD) activity, and a microsatellite stable (MSS) phenotype. In the absence of contraindications, the patient initiated first-line systemic chemotherapy on 3 December 2024, consisting of biweekly FOLFOX (fluorouracil, leucovorin, and oxaliplatin) in combination with panitumumab. After four cycles of treatment, in early March 2025, the patient developed acute left-sided ocular symptoms, including reduced visual acuity and ocular pain. Ophthalmologic evaluation, including ocular ultrasonography, revealed a choroidal lesion involving both the posterior and anterior segments, predominantly at the 12 o’clock meridian. A brain magnetic resonance imaging (MRI), performed on March 11, identified a 19 mm contrast-enhancing lesion at the superolateral aspect of the left globe, with no evidence of intracranial involvement (Figure 1). A restaging CT scan conducted 3 days later confirmed stable hepatic disease but documented a newly visible choroidal lesion measuring 18 × 8 mm (Figure 2). The patients gave informed consent for genetic assessments, article writing and publication. The study was approved by the IRB of the Centro AMES with protocol. n. CA06/2025.

Based on these findings, the patient underwent stereotactic radiotherapy to the orbital lesion using the CyberKnife® system. Between April 7 and April 11, a total dose of 22.5 Gy was delivered in five fractions to the 80% isodose line. At the time of this report, the patient has initiated second-line systemic therapy consisting of oral capecitabine, intravenous irinotecan, and bevacizumab, administered every 21 days.

## Genetic assessment

In parallel with clinical management, molecular profiling was expanded to explore the clonal dynamics of the malignancy. The primary tumor underwent next-generation sequencing (NGS) on formalin-fixed paraffin-embedded (FFPE) tissue. Briefly, following DNA extraction, libraries were prepared using the TruSight Oncology^®^ 500 kit, which targets 523 cancer-related genes (see Supplementary File S1 for the complete list). This assay enables the detection of multiple classes of genomic alterations, including single nucleotide variants (SNVs), insertions and deletions (indels), splice variants, copy number alterations, gene fusions, as well as immunotherapy-related biomarkers such as tumor mutational burden (TMB) and microsatellite instability (MSI). Sequencing was performed on the Illumina NovaSeq 6000 platform (San Diego, USA). Variant classification was performed according to the 2017 joint consensus recommendations of the American College of Medical Genetics and Genomics (ACMG) and the Association for Molecular Pathology (AMP) (Richards et al., 2015). No Tier IA variants were detected. A single Tier IB pathogenic alteration was identified in *TP53* (p.E286K), along with four Tier IIC variants affecting *BRCA2* (p.D1420Y), *FGFR4* (p.G388R), *MSH6* (p.V878A), and a truncating mutation in *RAD50* (p.Y625*). Additional Tier IID findings included missense or truncating alterations in *CCND3* (p.S259A), *MAGI2* (p.R766*), and *PDK1* (p.R144Yfs*18), as well as copy number gains involving *LAMP1*, *BRCA2*, and *FGF9* (each at three copies) and a copy number loss in *FGFR3* (one copy). The TMB was estimated at 7.1 mutations per megabase, and the tumor was MSS.

Prior to the initiation of ocular radiotherapy, a second molecular assessment was performed on ctDNA using the same TSO500 platform. This analysis revealed a markedly more heterogeneous and evolved mutational landscape. Notably, two new Tier IA events, absent in the primary tumor, were identified: an *EGFR* copy number gain (three copies) and a somatic *JAK2* p.V617F mutation. In addition, the ctDNA profile demonstrated the acquisition of multiple new Tier IIC and IID variants (Table 1). The ctDNA TMB had substantially increased to 44.9 mutations per megabase, although the MSS phenotype was maintained. Anonymised sequencing data from tumour tissue and liquid biopsy have been deposited in the European Nucleotide Archive (ENA) (Supplementary File S2).

**TABLE 1:** Genetic comparison between the primary tumor and the metastatic tumor at the time of choroidal metastasis development.

	Primary tumor	Metastatic tumor
Sequencing method	TSO500 on FFPE tissue	TSO500 on ctDNA
Variants		
Tier IA variants	None	*EGFR* copy gain (3 copies) *JAK2* p.V617F
Tier IB variants	*TP53* p.E286K	*TP53* p.E286K (persisted)
Tier IIC variants	*BRCA2* p.D1420Y *FGFR4* p.G388R *MSH6* p.V878A *RAD50* p.Y625*	Persistence of primary variants plus new: *NRAS* p.G12D *ARID1B* p.G721Afs11 *AXIN1* p.K641Rfs64 *APC* p.E1309Dfs12 *CUX1* p.P782Rfs26 *DNMT3A* p.A571Lfs80 *POLD1* p.E346Sfs47 *PRKDC* p.R2703Gfs13 *SETD2* p.R1407Gfs5, p.K787* *SMO* p.P694Lfs82 *TP53* p.P72R Copy gains: *BRAF*, *CDK6*, *FGF6*, *FGFR2*, *BRCA2* (now 4 copies) Copy loss: *BRCA1*
Tier IID variants	*CCND3* p.S259A *MAGI2* p.R766* *PDK1* p.R144Yfs*18 Copy gains: *LAMP1*, *BRCA2*, *FGF9* (3 copies) Copy loss: *FGFR3* (1 copy)	Persistence of primary variants plus new: *ARID5B* p.K1027Rfs8 *ASXL1* p.L815P *ERBB2* p.P1170A *FLT4* p.P30Rfs3 *GLI1* p.P567Lfs46 *MYCN* p.P45Rfs86 *NOTCH2* p.S1419Afs8 *PAX5* p.A322Lfs11 Copy gains: *FGF14*, *FGF9* (4 copies), *LAMP1* (4 copies) Copy losses: *FGFR3*, *FGF7* (1 copy each)
Tumor Mutational Burden	7.1 mutations/mb	44.9 mutations/mb
Microsatellite Status	MSS	MSS

ct: circulating tumor; FFPE: formalin-fixed paraffin-embedded; Mb: mega bases; MSS: microsatellite stable; TSO500: TruSightOncology^®^ 500 panel.

## Discussion

This case illustrates an exceptional metastatic trajectory in CRC, culminating in choroidal dissemination, a phenomenon reported in fewer than 1% of metastatic CRC cases. Importantly, the availability of longitudinal genomic profiling has allowed us to map clonal shifts potentially correlating with this rare phenotype. The primary tumor was sequenced using the TSO500 panel on FFPE tissue, capturing a moderately altered molecular landscape, characterized by canonical CRC drivers such as *TP53* (p.E286K) and additional mutations of uncertain or emerging relevance, including in *BRCA2*, *FGFR4*, *MSH6*, and *RAD50*. This initial profile, with a TMB of 7.1 mutations per megabase and confirmed MSS, suggested a genomically stable phenotype consistent with conventional colorectal adenocarcinoma (Hecht et al., 2023; San-Román-Gil et al., 2023). The subsequent liquid biopsy, obtained from plasma prior to ocular radiotherapy, uncovered a substantially more heterogeneous and evolved mutational profile. The ctDNA analysis, performed with the same TSO500 platform, not only confirmed the persistence of key founder mutations but also revealed multiple newly acquired alterations, indicating extensive clonal diversification. This genomic shift between the primary and metastatic compartments, likely reflecting both temporal tumor evolution and selective pressure imposed by therapy. Among these, the detection of *EGFR* amplification and a somatic *JAK2* p.V617F mutation, both classified as Tier IA, was particularly noteworthy. Both are classified as Tier IA, conferring potential biological and therapeutic relevance.

*EGFR* amplification has been implicated in resistance to anti-EGFR therapies and may contribute to tumor proliferation, angiogenesis, and organotropism, potentially facilitating ocular dissemination via choroidal vasculature (Benli et al., 2025). The *JAK2* p.V617F mutation, typically associated with myeloproliferative neoplasms, has been increasingly recognized in solid tumors (Jeong et al., 2008) and may mediate immune evasion, inflammation-driven metastasis, and niche adaptation (Wolf et al., 2012; Wu et al., 2017; Looyenga et al., 2012). The Janus kinase (JAK) family (comprising JAK1, JAK2, JAK3, and TYK2) plays a central role in cytokine-mediated signal transduction. Upon ligand binding to type I and type II cytokine receptors, JAKs become activated through trans-phosphorylation and, in turn, phosphorylate specific tyrosine residues on the receptor cytoplasmic domains. These phosphotyrosine motifs serve as docking sites for Signal Transducers and Activators of Transcription (STAT) proteins, which are subsequently phosphorylated by JAKs, dimerize, and translocate to the nucleus to regulate gene expression. The JAK-STAT pathway governs critical biological processes including proliferation, survival, differentiation, and immune modulation (Wang et al., 2024). Aberrant activation of JAK signaling, either via activating mutations (e.g., *JAK2* p.V617F) or cytokine dysregulation, can promote oncogenesis, immune escape, and metastatic competence in immunoprivileged niches (such as the choroid) (Galassi et al., 2021; Chen and Song, 2022) and is a potential therapeutic target (Liu et al., 2015; Wang et al., 2014; Park et al., 2019).

While *EGFR* amplification is a well-recognized mechanism of resistance to anti-EGFR therapies and its emergence in this patient may reflect selective therapeutic pressure, the identification of the *JAK2* p.V617F mutation in ctDNA raises more complex questions. This mutation, classically associated with myeloproliferative neoplasms, has been sparsely documented in solid tumors, and its presence in CRC remains highly atypical. Indeed, large-scale sequencing efforts encompassing thousands of CRC specimens have not identified *JAK2* p.V617F mutations in tumor tissue, suggesting that this alteration is exceedingly rare or potentially absent from primary colorectal carcinogenesis (Herreros-Villanueva et al., 2010; Strickler et al., 2018).

Several hypotheses may account for its detection in the present case. First, a sampling bias inherent to most molecular studies, wherein sequencing is largely performed on primary tumor tissues, may obscure events specific to metastatic outgrowths, especially those within immune-privileged sites like the choroid. From this perspective, *JAK2* p.V617F could represent a metastatic driver that has been under-recognized due to its inherently low incidence and the limited sampling of metastatic lesions in the current literature (Strickler et al., 2018). Second, the mutation may not originate from the tumor at all, but instead reflect a hematopoietic clone of indeterminate potential (CHIP), a phenomenon increasingly recognized in the aging population (Natarajan, 2023). Indeed, *JAK2* p.V617F is one of the most prevalent mutations found in clonal hematopoiesis and has been reported in approximately 0.2% of the general population, especially with advancing age. This alternative source would imply a false-positive tumor signal, not uncommon in ctDNA-based analyses, particularly when matched white blood cell DNA is not sequenced in parallel to exclude CHIP. A third and more speculative possibility is that *JAK2* p.V617F, though exceedingly rare, may represent a legitimate yet infrequent event in the molecular evolution of metastatic CRC. Some recent data suggest that this mutation can occur in solid malignancies beyond hematopoietic cancers, potentially contributing to stromal remodeling, immune escape, or organotropic metastasis (Zhao et al., 2020). In this framework, its presence in ctDNA could denote a biologically significant subclonal event within a metastatic niche, possibly contributing to dissemination toward less common sites such as the choroid, through modulation of the JAK-STAT pathway and associated inflammatory circuits.

Although a baseline ctDNA sample is lacking for temporal resolution, the stark contrast between the primary tumor’s molecular profile and the subsequent liquid biopsy suggests that *EGFR* amplification and *JAK2* p.V617F emerged during disease progression, possibly as a consequence of therapy-induced selective pressure or metastatic adaptation. However, the absence of a site-specific biopsy and matched normal sequencing limits our ability to definitively assign the origin of the *EGFR* amplification and *JAK2* p.V617F signals. Nonetheless, these findings highlight the interpretative complexity of liquid biopsy in advanced cancer and underscore the importance of integrating molecular data with clinical and radiologic context. In this case, the molecular shift coincided with a phenotypically unusual metastatic trajectory, reinforcing the notion that clonal evolution may yield unexpected yet biologically meaningful adaptations beyond canonical CRC paradigms. In fact, while ctDNA captures a cumulative signal from multiple disease sites, the consistent MSS status and the marked increase in TMB (from 7.1 to 44.9 muts/Mb) support an ongoing clonal diversification under therapeutic pressure.

Furthermore, several mutations identified after first-line therapy, including alterations in *NRAS*, *POLD1*, and key components of the Wnt signaling pathway (*APC*, *AXIN1*, and *CUX1*), warrant further consideration in the context of metastatic tropism and therapeutic resistance. Activating *NRAS* variants, although less frequent than *KRAS*, have been increasingly implicated in resistance to anti-EGFR therapies through reactivation of downstream MAPK and PI3K signaling pathways (Zhou et al., 2021). The emergence of NRAS mutant subclones in this patient may therefore represent a plausible mechanism of acquired resistance to EGFR-targeted agents. Mutations in *POLD1*, which encodes the catalytic subunit of DNA polymerase δ responsible for proofreading during DNA replication, have been associated with ultra-hypermutated phenotypes and elevated TMB in CRC (Xing et al., 2022). Such alterations can drive genomic instability and clonal diversification, potentially accelerating the development of resistant subpopulations under therapeutic pressure. Furthermore, of particular relevance are alterations in genes involved in the Wnt signaling axis. Mutations in *APC*, *AXIN1*, and *CUX1* are known to promote tumor aggressiveness, stemness, and metastatic dissemination. *APC* is the most frequently mutated gene in colorectal carcinogenesis, acting as a gatekeeper of the Wnt pathway by regulating β-catenin degradation (Zhao et al., 2022). AXIN1, a core component of the β-catenin destruction complex, although less commonly mutated, similarly contributes to aberrant Wnt pathway activation (Qiu et al., 2024). CUX1, a homeodomain transcription factor, exerts a context-dependent oncogenic role: its overexpression may support cell proliferation, metastasis, and DNA repair capacity, particularly in cooperation with activated RAS signaling, while its haploinsufficiency has been linked to increased chromosomal instability and impaired genome integrity (Liu et al., 2020). These genomic alterations, while diverse in nature, collectively highlight the intricate molecular landscape of metastatic CRC and illustrate how multiple, converging pathways may cooperate to shape aggressive phenotypes and enable tumor adaptation within immune-privileged environments such as the choroid.

The second genetic assessment in the case described was performed using liquid biopsy and ctDNA analysis. It is important to emphasize that although ctDNA offers distinct advantages, such as faster turnaround time, improved safety, and the ability to capture tumor heterogeneity, current international guidelines still advocate for a complementary approach in clinical practice. In particular, the European Society for Medical Oncology (ESMO) (Pascual et al., 2022) and the American Society of Clinical Oncology (ASCO)/College of American Pathologists (CAP) (Merker et al., 2018) recognize ctDNA genotyping as a valuable tool in advanced disease settings, especially when tumor tissue is unavailable, insufficient, or when rapid therapeutic decisions are required. However, ctDNA analysis should not yet be considered a substitute for tissue sequencing. Instead, an integrated strategy, combining tissue-based NGS at diagnosis with serial ctDNA analysis during treatment and disease progression, offers a more comprehensive and dynamic view of tumor evolution, enhancing diagnostic accuracy while mitigating the limitations inherent to each individual method.

This case highlights the utility of integrating tissue- and plasma-based NGS for monitoring tumour evolution in CRC, and underscores the relevance of high-resolution genomic profiling in potentially uncovering the molecular mechanisms underlying rare metastatic patterns.

## Data Availability

The datasets presented in this study can be found in online repositories. The names of the repository/repositories and accession number(s) can be found below: https://www.ebi.ac.uk/ena, ERR14988467 https://www.ebi.ac.uk/ena, ERR14988468.
