# Supplementary material for: Genomic trajectories of colorectal cancer with choroidal metastasis: longitudinal insights from tissue and liquid biopsy via next-generation sequencing
Source: Front Genet. 2025 Aug 6;16:1632580. doi: 10.3389/fgene.2025.1632580 (PMC12364847; doi:10.3389/fgene.2025.1632580)
Supplement: Supplementary file 1 [file DataSheet2.pdf]

Supplementary File 2

Deposition of patient sequence data in the European Nucleotide Archive (ENA)

The clinical case is described in "Genomic trajectories of colorectal cancer with choroidal metastasis: longitudinal insights from tissue and liquid biopsy via next-generation sequencing."

https://www.ebi.ac.uk/ena/browser/home

Accession	Instrument	Study	Sample	Experiment	Sample accession	Biosample
ERR14988467	Illumina NovaSeq 6000	ERP172572	ERS24509814	ERX14392724	ERS24509815	SAMEA118323784 (tissue)
ERR14988468	Illumina NovaSeq 6000	ERP172572	ERS24509815	ERX14392725	ERS24509814	SAMEA118323783 (liquid biopsy)
